# Examining the protective influence of posttraumatic growth on interpersonal suicide risk factors in a 6-week longitudinal study

**DOI:** 10.3389/fpsyg.2022.998836

**Published:** 2022-10-20

**Authors:** Meryem Betul Yasdiman, Ellen Townsend, Laura E. R. Blackie

**Affiliations:** ^1^Personality, Social Psychology and Health Research Group, University of Nottingham, Nottingham, United Kingdom; ^2^Self-Harm Research Group, University of Nottingham, Nottingham, United Kingdom

**Keywords:** posttraumatic growth, suicide, burdensomeness, thwarted belongingness, longitudinal design

## Abstract

Research has found an inverse relationship between posttraumatic growth (PTG) and suicidal ideation in military and community samples that holds when controlling for other suicide risk factors. However, further research is needed into the underlying mechanisms to clarify how PTG protects against the formation of suicidal ideation. The current two-wave longitudinal study examined whether perceiving PTG from recent adverse circumstances while in a national lockdown during the COVID-19 pandemic attenuated the positive relationship of two interpersonal suicide risk factors – perceived burdensomeness (PB) and thwarted belonginess (TB)–over 6 weeks. Participants (*n* = 170) were recruited online from Prolific from income-deprived areas in the United Kingdom (mean age = 37.65; *SD* = 12.50; 53.5% female). *Post-hoc* power analyses indicated we had insufficient power to examine the hypothesised mediation for TB. We examined whether PTG mediated the relationship between PB at wave 1 and wave 2 while controlling for depression and anxiety in a sample of individuals at-risk for suicidal ideation. PTG did significantly and partially mediate the positive relationship between PB at wave 1 and 2. We discuss the theoretical and clinical implications that could result if future research successfully replicates these initial exploratory findings.

## Introduction

Posttraumatic growth (PTG) is the positive psychological changes individuals may report in their identity, relationships, and worldviews after struggling with distressing, and potentially traumatic life experiences (Tedeschi and Calhoun, 2004). It is typically measured *via* self-report questionnaire, asking individuals to rate positive changes experienced in personal strength, spirituality, appreciation of life, personal relationships, and identification of new possibilities in life (Tedeschi and Calhoun, 1996). Although this methodology is retrospective and might not mirror how individuals change in these dimensions over time (Frazier et al., 2009), the act of perceiving PTG from past adversity could have an adaptive role in helping individuals cope with current stressors (Tennen and Affleck, 2002). Indeed, researchers have started to examine whether PTG is a protective factor against the development of suicidal ideation due to calls to explore suicide resiliency (Wingate et al., 2006). Bush et al. (2011) found an inverse relationship between PTG and suicidal ideation in a correlational study with US army personnel when controlling for risk factors, such as depression, PTSD, and combat exposure. Similarly, Gallaway et al. (2011) found this inverse relationship between PTG and suicidal ideation in US army soldiers. This relationship between PTG and suicidal ideation has also been observed in non-military populations (Yu et al., 2010; Sheline, 2013; Yasdiman et al., 2022), indicating that PTG could have a protective role in this context.

However, although these findings are promising, research into the underlying mechanisms is needed to understand how PTG protects against the formation of suicidal ideation. Recent research by Yasdiman et al. (2022) has started to address this question by exploring how PTG fits into the Integrated Motivational-Volitional (IMV) Model of Suicidal Behaviour (O'Connor and Kirtley, 2018). The IMV is an established model of suicide behaviour mapping the potential pathways between risk factors to suicidal ideation and behaviours. In a pre-registered design and with a well powered community sample, Yasdiman et al. (2022) found that contrary to predictions derived from the IMV model, PTG did not moderate the relationship between defeat and entrapment or between entrapment and suicidal ideation. Yet, Yasdiman et al. (2022) did find the inverse relationship between PTG and suicidal ideation. The current study builds on this research to examine whether PTG has a protective function on suicidal ideation indirectly by attenuating some of the interpersonal suicide risk factors that increase feelings of entrapment and strengthen the IMV pathway between entrapment and suicidal ideation (O'Connor and Kirtley, 2018).

### Theoretical rationale for current study

The IMV model (O'Connor and Kirtley, 2018) outlines a pathway from distressing experiences to feelings of defeat, to feelings of entrapment, to suicidal ideation. The model outlines how after a distressing experience an individual may feel overwhelmed by negative thoughts (i.e., defeated) and may have perceptions of being unable to change their circumstances (i.e., entrapment), thereby increasing their risk of suicidal ideation. The IMV model also identifies risk factors that strengthen these pathways and increase the risk of suicidal ideation, and protective factors that weaken these pathways and reduce the risk of suicidal ideation. Of relevance to the current study, two interpersonal risk factors–perceived burdensomeness (PB) and thwarted belongingness (TB) - have been proposed in the IMV model to strengthen the pathway between entrapment and suicidal ideation. It should be noted that negative interpersonal thoughts were first introduced by Joiner (2005) in the Interpersonal Theory of Suicide as direct pathways to suicidal ideation and were added as motivational moderators in the IMV model in 2011. PB is defined as perceiving oneself as ineffective and incompetent, which leads to the feeling of being a liability to other people (Joiner, 2005). TB is defined as an unmet need to belong, resulting in failed social connectedness (Van Orden et al., 2012).

Research that has examined these two risk factors within the context of suicidal ideation and behaviours (e.g., Forkmann and Teismann, 2017; Levi-Belz and Aisenberg, 2021) has shown the importance of their inclusion when assessing suicide risk; and as theorised by the IMV model, researchers have found PB and TB moderate the pathway between entrapment and suicidal ideation (Lucht et al., 2020). Recently, Blevins (2019) examined the association between PTG and these two suicide risk factors in a correlational study with a military personnel population and found negative associations between PTG and both PB and TB. Considering findings from Blevins (2019) and the findings from Yasdiman et al. (2022) showing PTG did not moderate key pathways within the IMV model, we propose that PTG could mitigate the development of suicidal ideation indirectly through attenuating interpersonal risk factors.

### Current study design and hypotheses

**Figure 1:**
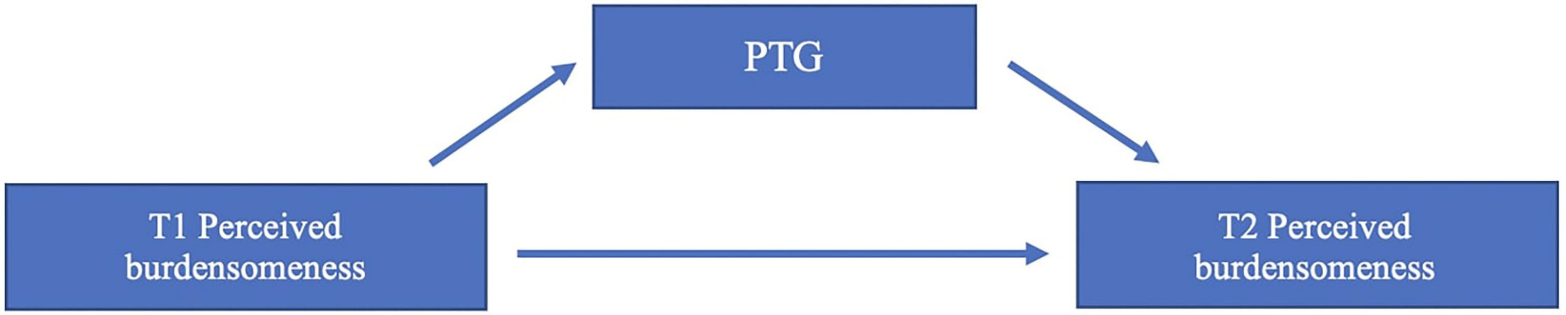
PTG mediating the relationship between perceived burdensomeness (PB) at T1 and perceived burdensomeness (PB) at T2. Anxiety and depression were considered as covariates.

**Figure 2:**
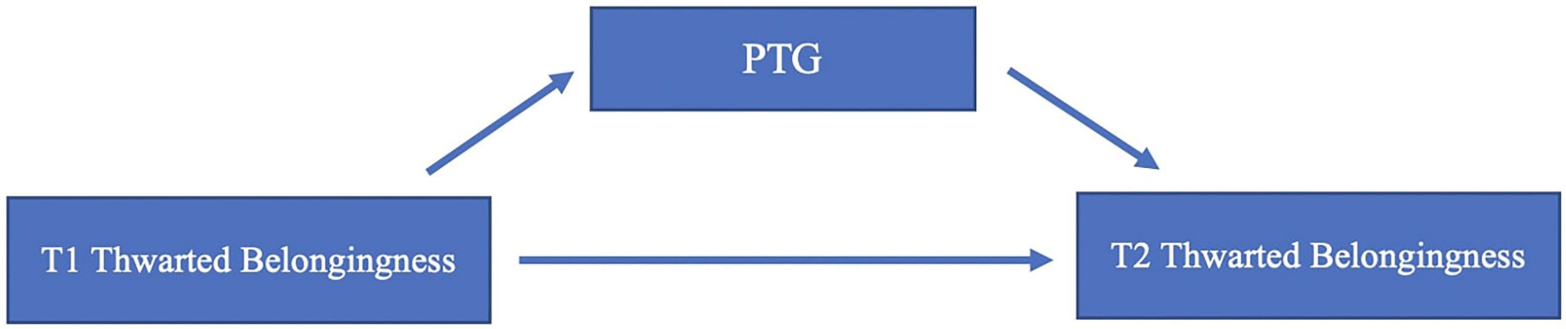
PTG mediating the relationship between thwarted belongingness (TB) at T1 and thwarted belongingness (TB) at T2. Anxiety and depression were considered as covariates.

Although researchers have examined the IMV model cross-sectionally (e.g., Yasdiman et al., 2022), it is a model that outlines the temporal dynamics between key processes that increase individuals’ risk of suicide behavior over time (O’Connor, 2011; O'Connor and Kirtley, 2018). In this study, we examined whether perceiving PTG from recent distressing experiences would reduce the levels of PB and TB experienced by individuals over a period of 6-weeks, which theoretically, would weaken the relationship between these interpersonal risk factors and entrapment and thereby weaken the relationship between entrapment and suicidal ideation. We examine whether PTG mediates the relationship between PB at wave 1 and wave 2 (Figure 1), and between TB at wave 1 and wave 2 (Figure 2) while controlling for depression and anxiety (Dhingra et al., 2016) in a sample of individuals at-risk for suicidal ideation. We expect wave 1 PB (and TB) scores to correlate positively with their own respective wave 2 scores, but for this relationship to be reduced when PTG is included as a mediator. We used a short-term longitudinal study (6 weeks) because Ribeiro et al. (2016)) emphasised the need for studies with shorter timeframes from their meta-analysis of longitudinal studies on suicidality.

We examined whether perceiving PTG from recent adverse circumstances while in a national COVID-19 lockdown reduced PB and TB across 6-weeks within an at-risk population of individuals living in economically deprived areas in the United Kingdom. A similar recruitment strategy was used by Griffiths et al. (2014) who found over half of their participants reported clinically relevant levels of psychopathology, indicating that individuals who live in deprived areas are a clinically meaningful group and at increased risk of developing suicidal ideation. This group has been further identified as a vulnerable and at-risk group during the COVID-19 pandemic (Gratz et al., 2020; Raj et al., 2021), indicating the importance of research examining factors supporting suicide resiliency within this group.

## Materials and methods

We pre-registered the design, hypotheses, and data analytic plan on OSF.^1^

However, we changed our hypotheses and adapted our data analytic plan accordingly after data collection. We had planned to examine the influence of PTG on moderators in the IMV model examining the relationship of PTG on PB and TB while controlling for baseline measures of PB and TB. On reflection, we consider PTG as an active process that might strengthen or weaken the influence of some IMV moderators on set pathways. This longitudinal study allowed us to test these hypotheses, albeit in a short timeframe, but within a context that reflects the temporal pathways specified in the IMV model. The analyses should therefore be considered exploratory and will require further replication.

### Design

This study employed a two-wave longitudinal design. Data was collected at wave 1 (T1) in October 2020 and 6-weeks later at wave 2 (T2) in December 2020 during national lockdowns in the COVID-19 pandemic. One hundred and seventy participants in some of the most income-deprived areas in the United Kingdom completed the T1 and T2 questionnaires.

### Participants

Two hundred and 12 participants aged 18 or older were recruited from income deprived areas in the United Kingdom^2^ according to the Ministry of Housing, Communities, and Local Government (2019) on Prolific and were financially compensated according to Prolific payment guidelines. The final sample consisted of 170 participants who completed both waves. The mean age of the sample was 37.65 (*SD* = 12.50, with an age range from 18 to 73). Most of the sample were female (*N* = 91; 53.5%), and 86.5% (*N* = 147) identified as White, 6.5% as Asian, 2.9% as Black, and 4.1% answered in the open-text box. Most of the sample were in full-time employment (*N* = 99, 58.2%), while 27 were in part-time employment (15.9%). The remaining participants chose ‘other’ or ‘unemployed and not seeking job’ options. The mean scores of T1 PB and TB were 11.25 (6.66) and 28.41 (12.82), respectively. The mean scores of these two interpersonal risk factors are comparable to Cero et al. (2015) within a sample of psychiatric in-patients (i.e., 11.73 (11.33) for PB and 24.07 (13.61) for TB) and higher than the means Cero et al. reported for an undergraduate sample (i.e., 2.06 (4.31) for PB and 10.63 (9.40) for TB). This suggests that participants in this sample were an at-risk group with high levels of interpersonal suicide risk factors of PB and TB.

### Procedure and questionnaires

Ethical approval was obtained from the authors’ School Ethics Committee prior to data collection (S1279). Participants were recruited *via* the United Kingdom Prolific website in exchange for financial compensation based on Prolific payment recommendations^3^. The T1 survey was completed online *via* a Qualtrics survey link through Prolific. This online survey contained the information sheet, consent form, and the T1 questionnaires (as outlined below). After 6 weeks, the same participants were invited to take part in the T2 Qualtrics survey *via* the Prolific website. At the end of both surveys, participants were presented with funny images to elicit a positive mood (Goritz, 2007), relevant United Kingdom support services, and the researchers’ contact information. For brevity we report only the questionnaires used in these analyses, but interested readers can review the full list of questionnaires administered on the OSF pre-registration URL.

*Demographic information:* Questions on age, gender, ethnicity, and employment status were collected.

*Interpersonal Needs Questionnaire:* This 15-item questionnaire assesses whether people are feeling a burden to others or experiencing a low sense of social belongingness (Van Orden et al., 2008) during the past few days. Example items from the burdensomeness (PB) sub-scale is ‘*These days, I think my death would be a relief to the people in my life*’ and one from the belongingness (TB) sub-scale is ‘*These days, I feel disconnected from other people*’. Participants answered each item on a seven-point scale ranging from: (1) ‘*Not at all true for me*’ to (7) ‘*very true for me’*. Cronbach’s Alpha for the PB scale was 0.94 and for TB was.93 at T1 (and 94 and.92, respectively at T2).

*Hospital Anxiety and Depression Scale (HADS):* This scale was used in the T1 survey to assess symptoms of anxiety and depression (Zigmond and Snaith, 1983) on a 4-point scale (from 0 to 3) during the past week with higher scores indicating greater symptoms of anxiety and depression. It consists of 14 questions, of which 7 correspond to the anxiety subscale, and 7 correspond to the depression subscale. Cronbach’s Alphas were.85 and.82 for anxiety and depression, respectively.

*Post-Traumatic Growth Inventory:* This measure was used within the T2 survey because our hypotheses are about the potential beneficial value of perceptions of PTG from adversity, rather than if genuine PTG manifests over time. Participants were first asked to think about a stressful life event that had happened to them recently, which did not have to be about COVID-19 pandemic specifically. They were given 15 s before proceeding to the next page. After that, they were presented with the 21-item PTGI measure and asked to answer questions referring to the recent stressor they thought about on the previous page. This scale assessed perceived positive changes as a result of stressful event on the following subscales: personal strength, appreciation of life, relating to others, new possibilities, and spiritual change (Tedeschi and Calhoun, 1996). Respondents answered each item on a 6-point scale, ranging from (0) ‘*I did not experience this change as a result of the event*’ to (5) *‘I experienced this change to a very great degree as a result of the event’*. An example item from the scale includes: *‘I have a greater appreciation for the value of my own life’*. Cronbach’s Alpha here was.94.

## Results

The analyses were conducted using SPSS 28 and the PROCESS macro plug-in on SPSS to test mediation (Hayes, 2017).

### Data preparation

Forty-two participants were either excluded (*n* = 14) or did not return to participate in wave 2 (*n* = 28). Participants were excluded if their ID at wave 1 and wave 2 could not be matched (*n* = 8) or if their completion rate was less than the *a priori* criterion of 75% of the full survey (*n* = 6; Wetherall et al., 2019). Item-level missing data was checked for each questionnaire, and there were no missing data issues. There were no significant differences in the mean age between the retained sample (*n* = 170; *M* = 37.67, *SD* = 12.44) and the excluded sample (*M* = 34.92, *SD* = 12.19), *t*(197) = 1.066, *p* = 0.14. Nor were there any significant differences between the samples in gender, χ^2^(1) = 0.315, *p* = 0.574.

### Power analyses

We ran a Monte Carlo post-hoc power analysis using the power estimator tool developed by Schoemann et al. (2020) because we deviated from our pre-registered data analytic plan, as previously justified in the method section. These analyses were to find the power we had to observe mediation when including the covariates and with our set sample size of *n* = 170. The analyses showed we were sufficiently powered at 0.83 to detect the H1 mediation involving PB, but insufficiently powered at 0.25 to detect the H2 mediation involving TB. For this reason, we conduct and report only the results for the H1 mediation.

### Descriptive statistics and correlations

Table 1 reports the mean (*M*), standard deviation (*SD*), and Pearson correlations between PTG, T1 PB, T2 PB, depression and anxiety using bootstrapping set at 1000 samples, which is a robust method to deal with skewness in the data (Field, 2017). PB at T1 and T2 were significantly positively correlated with large effect sizes, indicating that higher perceptions of burden at wave 1 were also associated with higher perceptions of burden at wave 2. PTG was positively associated with PB at T1 with a medium effect size and negatively associated with PB at T2 with a small effect size. PB at T1 and T2 were positively associated with depression and anxiety with large effect sizes. The effect size interpretations are based on Gignac and Szodorai (2016)‘s categories of effect size (small: 0.10, medium: 0.20, large: 0.30).

**Table 1:** Means, standard deviations, and correlations coefficients between PTG, depression, anxiety, and burdensomeness (waves 1 and 2).

Variable	1	2	3	4	5
1. PTG	–				
2. Perceived Burden1	0.226**[0.088, 0.350]	–			
3. Perceived Burden2	−0.155*[−0.284, −0.024]	0.528***[0.374, 0.684]			
4. Anxiety	0.207**[0.055, 0.357]	0.355***[0.219, 0.481]	0.311**[0.208, 0.425]	–	
5. Depression	0.008[−0.153, 0.177]	0.408***[0.274, 0.542]	0.381***[0.225, 0.528]	0.605***[0.493, 0.694]	–
*M*	35.00	11.68	10.17	7.81	5.84
*SD*	22.46	6.68	6.14	4.15	3.98
Range	0–105	6–42	6–42	0–21	0–21

*p < 0.05, **p < 0.01, ***p < 0.001, Perceived Burden1, Perceived burdensomeness at T1; Perceived Burden2, Perceived burdensomeness at T2. Bootstrapped confidence intervals are presented in brackets [lower CI, upper CI].

### Mediation analyses

Before examining whether PTG mediated the relationship between PB at T1 and T2, we examined whether the data met assumptions required for regression (e.g., outliers, normality and homoscedasticity). We did not identify any extreme cases that exceeded the cut-off scores when using a conservative strategy requiring cases to exceed cut-off scores of at least 2 or more of these outlier statistics: Mahalanobis distance, Cook’s distance and Leverage. Thus, no participants were excluded. We did identify violations to both the normality of the residuals and homoscedasticity assumptions. To correct for these issues, we followed recommendations made by Field (2017) and used the bootstrapping procedure and report the heteroscedasticity-consistent standard errors (HC4; Hayes and Cai, 2007) in the mediation.

**Figure 3:**
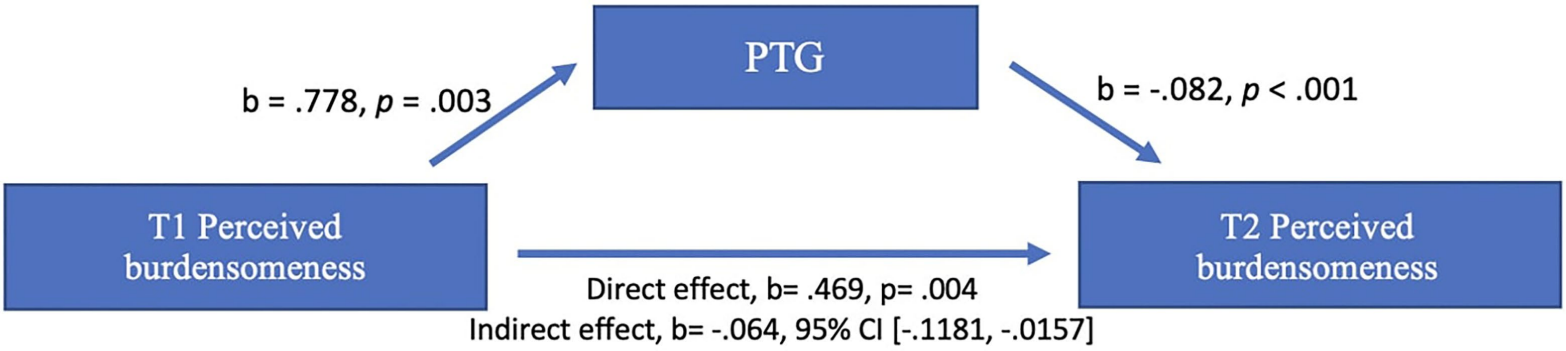
PTG mediating the relationship between T1 perceived burdensomeness and T2 perceived burdensomeness. Unstandardised regression coefficient (b) values are reported. Depression and anxiety were controlled in the analysis. The indirect effect is not associated with a *p* value (see Hayes, 2017).

We tested the H1 mediation using the PROCESS macro (Hayes, 2017; using model 4; 2000 resamples). We regressed PB at T1 (X) onto PTG (M) and PB at T2 (Y) entering anxiety and depression as covariates. As predicted, the direct effect of PB T1 on PB T2 (Figure 3) was significant and positive. The indirect pathway between PB T1 and T2, *via* PTG, was significant (indirect effect, *b* = −0.064, SE = 0.025, 95% BCa CI = [−0.1181, −0.0157]; Figure 3), therefore PTG did mediate the relationship between PB T1 and T2. As expected, the relationship between PB T1 and T2 remained significant (b = 0.405, *p* = 0.004, BCa CI = [0.1298, 0.6799]) when PTG was entered into the regression, indicating partial mediation.

## Discussion

We examined whether individuals in an at-risk group who perceived PTG from recent adversity while in a lockdown in the COVID-19 pandemic^4^ would report lower levels of interpersonal suicide risk factors over a period of 6-weeks. The results for one interpersonal suicide risk factor – PB – confirmed our hypotheses, as PTG (at T2) partially mediated the relationship between scores of PB over 6 weeks from T1 to T2. Thus, individuals who perceived higher levels of PTG from their recent experiences of adversity during the COVID-19 lockdown in the United Kingdom in 2020 had an attenuated relationship between PB scores across 6-weeks.

The direct association between PB at T1 and T2 was positive and significant, therefore, as expected, higher feelings of burdensomeness were associated with higher feelings of burdensomeness 6 weeks later. According to the IMV model of suicidal behaviour (O'Connor and Kirtley, 2018), higher levels of burdensomeness could serve to strengthen the pathway between entrapment and suicidal ideation. Thus, although a small effect, it is not insignificant to find that PTG attenuated the existing positive relationship of a key interpersonal suicide risk factor over time. This finding extends the correlational findings reported by Blevins (2019) between PB and PTG, and to our knowledge is the first study to examine this relationship longitudinally. Blevins (2019) documented an inverse relationship between TB and PTG as well, but unfortunately, we were too underpowered in this study to examine the hypothesis for TB. Future research is needed to first examine if this exploratory finding that we observed between PB and PTG can be replicated, and to examine if a similar finding is observed between PTG and TB.

### Implications and future directions

Our findings are consistent with existing research on this topic and expand knowledge in this area. First, we found an inverse relationship between PTG and key interpersonal risk factors for suicide that have been previously documented in correlational cross-sectional research (Blevins, 2019). Second, our short-term longitudinal study expanded the current research by observing that perceiving PTG from recent adversity could attenuate the existing positive association between feelings of burdensomeness over 6 weeks. This builds on recent work by Yasdiman et al. (2022) who had observed PTG did not moderate key pathways to suicidal ideation in the IMV model, but it did correlate directly and negatively with suicidal ideation. Through the lens of the IMV model, the results of this study suggest that PTG may exert a protective function not by influencing key pathways between defeat and entrapment or entrapment and suicidal ideation, but instead by indirectly influencing other motivational phase moderators (i.e., risk factors), such as PB that strengthen the pathway between entrapment and suicidal ideation. We note that this study focused only on whether PTG reduced the saliency of these two risk factors. It did not measure entrapment or suicidal ideation. Future research would be needed to examine the longitudinal associations between PTG, interpersonal risk factors (PB and TB), entrapment, and suicidal ideation.

In this study, we found that greater perceptions of PTG weakened the relationship between PB over 6-weeks, but we did have scope to examine the causes for why some individuals experienced more PTG than others. Our finding is consistent with some past research documenting a positive relationship between PTG and distress (e.g., Shakespeare-Finch and Lurie-Beck, 2014; Zhen and Zhou, 2022), suggesting that perceiving PTG might be a coping strategy that individuals can use when trying to manage high levels of distress from adverse experiences. However, we should note that the association between distress and PTG is inconsistent, with some researchers finding a positive association (e.g., Dekel et al., 2012) and others finding a negative association (e.g., Frazier et al., 2001) therefore further research is needed to examine the relationship between the severity of distress and PTG within the context of suicide models.

Although these initial findings should be viewed as exploratory given the deviation from our pre-registered hypotheses (as previously justified in the method section), the findings have some important implications for research and suicide prevention strategies, if replicated in future research. Perceptions of burdensomeness are a robust predictor of suicidal ideation and suicide attempts controlling for other risk factors, such as depression and hopelessness (Van Orden et al., 2006; Hill and Pettit, 2014). Suicide models, however, suggest that perceptions of burdensomeness are a modifiable risk factor (Joiner, 2005; O'Connor and Kirtley, 2018) and are not stable and will vary over short time periods (Kleiman et al., 2017), in which case if PTG does exert a robust protective function on reducing burdensomeness, then it might inform psychosocial interventions to reduce suicidal ideation. For example, some aspects of PTG can be integrated into existing therapies (e.g., cognitive-behavioural therapy or emotion regulation psychotherapy; see Roepke et al., 2018) and these therapies have been found to be effective in decreasing the risk for self-harm repetition over time (Witt et al., 2021), which is a known risk factor for suicide (Hawton et al., 2003; Guan et al., 2012).

### Limitations

Despite the promising nature of our findings, there are some limitations and caution should be taken until these initial exploratory findings are replicated. We collected our data during the ongoing COVID-19 pandemic and resulting national lockdowns, so the relationships need to be examined in a non-pandemic climate to assess the generalisability of results. We asked our participants to report PTG based on a recent challenging experience. It might be quite challenging for individuals to perceive and identify PTG when in the midst of managing recent experiences of adversity. Although there is some longitudinal evidence demonstrating that individuals report increases in their perceptions of PTG across a short time period (e.g., 4–5 weeks; Danhauer et al., 2013), more research is needed to examine how perceptions of PTG change and the beneficial versus harmful consequences of perceptions of PTG over time. Relatedly, this was a short-term longitudinal study with only two waves of data, and therefore longitudinal research with three or more time points would provide greater insight into how perceptions of PTG change over time and how these changes in perceived PTG may influence interpersonal suicide risk factors, such as PB. In addition, 86.5% of our participants were identified their ethnicity as White, which may limit the generalisability of the results to the other people identified with different ethnicities. Despite these limitations, our study did find a potentially important result regarding the protective influence of PTG against suicidal ideation in a sample of participants living in some of the most income-deprived areas and at greater risk of suicide (Griffiths et al., 2014). Thus, if future research does replicate these findings, there could be significant advancements in researchers’ understanding of suicide resiliency that could inform clinical interventions.

## Data availability statement

The datasets presented in this study can be found in online repositories. The names of the repository/repositories and accession number(s) can be found at: Open Science Framework; https://osf.io/mpcuw/?view_only=a5d22fc8b2c4473e92abf2dce67796e9.

## Ethics statement

The studies involving human participants were reviewed and approved by University of Nottingham, School of Psychology Ethics Committee. The patients/participants provided their written informed consent to participate in this study.

## Author contributions

MY, LB, and ET: study design, interpretation of data, drafting of manuscript, and revisions. MY: data collection and analysis. All authors contributed to the article and approved the submitted version.

## Conflict of interest

The authors declare that the research was conducted in the absence of any commercial or financial relationships that could be construed as a potential conflict of interest.

## Publisher’s note

All claims expressed in this article are solely those of the authors and do not necessarily represent those of their affiliated organizations, or those of the publisher, the editors and the reviewers. Any product that may be evaluated in this article, or claim that may be made by its manufacturer, is not guaranteed or endorsed by the publisher.

## Footnotes

^1^ https://osf.io/qhpd9/?view_only=b9a5783098ec4207b7a7d63043247232

^2^These were some areas within Middlesbrough, Liverpool, Knowsley, Kingston upon Hull, Manchester, Blackpool, Birmingham, Burnley, Blackburn with Darwen, and Hartlepool.

^3^At the time of the data collection (in 2020), the minimum amount Prolific researchers could pay participants was £5. We compensated participants with an average of £6.62 and £6.41 per hour for T1 & T2 participation, respectively.

^4^We would like to note here that when responding to the PTG questions, participants were asked to consider a recent stressor; however, they were not asked to consider a stressor that was connected to COVID-19. People might have thought about COVID-19-related stressful events, such as lockdowns. However, they were not asked to reflect on the pandemic-related stressors. We wanted to draw attention to the fact that the study was performed during a pandemic, which may increase vulnerability to suicide risk.
